# Natural Bioactive Substances in Fruits of *Aronia melanocarpa* (Michx.) Elliott Exposed to Combined Light-Type, Chitosan Oligosaccharide, and Spent Mushroom Residue Treatments

**DOI:** 10.3390/plants12030604

**Published:** 2023-01-30

**Authors:** Yadong Duan, Xin Wei, Wenbo Zhao, Jinxia Li, Guang Yang, Shuang Zhou, Chunwei Zhou, Lei Zhang, Pengju Li, Shuai Hou, Deshan Shi, Cheng Liu, Baitao Guo

**Affiliations:** 1 Institute of Rural Revitalization Science and Technology, Heilongjiang Academy of Agricultural Sciences, Harbin 150028, China; 2 Northeast Institute of Geography and Agroecology, Chinese Academy of Sciences, Harbin 150081, China; 3 Huma Cold Temperate Zone Experimental Station of Conservation and Utilization of Wild Plant Germplasm Resources, Huma 165000, China; 4 Liaoning Institute of Pomology, Yingkou 115009, China; 5 College of Landscape Architecture, Northeast Forestry University, Harbin 150040, China; 6 Heilongjiang Greater Khingan Mountains Region Agriculture Forestry Research Institute, Jagedaqi 022450, China

**Keywords:** black chokeberry, nature-based solution, non-wood forest product, spent mushroom residue, chitosan oligosaccharide

## Abstract

Greenhouse culture is a practical approach to obtain non-wood forest products from berry fruit at a higher efficacy than resource silviculture in natural understory. In this study, three-year old black chokeberry (*Aronia melanocarpa* (Michx.) Elliott 1821) seedlings were transplanted to a greenhouse where sunlight was complemented by red- (69.4% red, 30.2% green, 0.4% blue) and blue-color (15.3% red, 64.9% green, 19.8% blue) light-emitting diode (LED) illuminations. Half of the planting soils were amended by spent mushroom residue (SMR) (not amendment as the control) and half the seedlings were sprayed by chitosan oligosaccharide (CO) on leaves. All treatments can increase seedling height, but only blue light reinforces the basal diameter growth. Compared to sunlight, exposure to blue light can promote leaf nitrogen and phosphorus concentrations, superoxide dismutase activity, and fruit proanthocyanidin content. The combination with CO addition will further increase chlorophyl a content, acid phosphatase activity, and total phenolics in fruit. SMR amended can induce the steady state uptake of nutrients but failed to impact fruit quality. Overall, we recommend the combination of blue light LED illumination plus CO addition to culture black chokeberry for the purpose to gain natural bioactive compounds.

## 1. Introduction

The sustainable development goal 15 was themed Life on Land, where oxygen, weather regulation, crop pollination, natural products, and ecosystem services are provided by nature [1,2,3]. Forests are a big tank to harbor species, but over 75% of biodiversity is being lost or reduced in terrestrial ecosystems [4,5]. Non-wood forest products (NWFPs) account for a major proportion of understory forest biodiversity [6]. The sustainable use of the NWFP resources can alleviate the stress of heavy exploitation and ensure the establishment of a cycle of supply-and-demand.

Introduced NWFP resources were advocated to be cultured at the understory layer of forests that were screened or managed to equip the objective environment. Meteorological factors have a joint driving effect on bioactive products in forest plants [7,8,9,10]. Forests are a natural nursery where NWFP resources can be managed to harvest natural products [11,12]. Natural bioactive products extracted from NWFP plants can be not only derived from human/industry-based accesses, but also by nature based solutions [13]. The management of NWFP plants in original or introduced habitats is a nature-based solution, which endorses a significant yield of bioactive compounds by managing stand attributes [11,14,15]. The greenhouse is a combined human/industry- and nature-based solution to culture NWFP plants with all environmental factors controlled [16,17,18,19]. The greenhouse cultural protocol is a critical precondition to successfully derive natural products from NWFP plants by controlling objective factors.

Transmittance of sunlight for NWFP populations can be adjusted by adjusting canopy gap [20,21], which matters for the yield of bioactive production [22]. Light quality can be determinative in medicinal or edible uses of NWFPs [23,24,25]. Sunlight spectrum can hardly be changed in natural forests [12,26,27]. Light emitting diode (LED) is an instrument that provides artificial illumination for greenhouse plants. It is flexible to change the spectra of LED light by adjusting the composition of wavelengths in red, green, and blue lights [19,25,28,29,30]. The effect of different types of LED spectra on bioactive compounds in NWFP plants is highly species-specific [23,24,31,32]. Roots and leaves are alternative sources and sinks to assimilate nutrient and light resources, respectively [25,33,34]. Hence, the effect of light spectrum on bioactive compound production is a result of the interaction between light and nitrogen (N) and phosphorus (P) nutrition, but relevant evidence is scarcely testified on NWFP plants in a greenhouse. 

Conventional field protocol employs a remarkable labor input to control weeds and implement fertilization for the culture of NWFP plants [35,36]. Herbicide and controlled-release fertilizer (CRF) were suggested as solutions, but they present threats in terms of environmental safety affecting underground water quality [37,38]. Soil amendment using organic materials is a newly strategy that can improve soil porosity and increase availability [39,40]. Fresh spent mushroom residue (SMR) is a recommended organic amendment due to its purified composition, enriched nutrient residue, and wide accumulations [17,41,42]. SMR has been well identified to be an environment-friendly replacement of peat in growth substrates for juvenile plant [43,44,45]. The employment of soil amendment using SMR has a potential to earn dual wins in greenhouse plant breeding by controlling the labor input and reducing the environment risk of solid pile of SMR. 

Plant bioactive compounds are synthesized through secondary metabolisms when botanic perception is signaled under a stressful condition [46,47]. Stressful condition also limits the ordinary process of plant growth and development, which is a contradiction against bioactive production [47]. For example, some LED spectra can benefit growth, nutrient uptake, and carbohydrate metabolism, but can also reduce the accumulation of total flavonoids and saponins in *Allium victorialis* [23]. As a responsive strategy, plants upregulate the syntheses of phytochemical compounds, such as oligosaccharides, to counter perceived stress [48,49]. Exposure to exogeneous oligosaccharide additives can also strengthen resistance to stress [50]. Oligosaccharides can be derived from enzymatic-hydrolyzed products of chitosan, which is produced from marine chitin through deacetylation [51]. Chitosan oligosaccharide (CO) can be widely obtained from seafood wastes and used as a plant-growth regulator. The direct addition of CO can increase drought assistance by controlling stomatal conductance in Buddhist pine (*Podocarpus macrophyllus*) seedlings [52]. The CO addition can also improve the ability of nutrient uptake and utilization in Buddhist pine [29,51] and fragrant rosewood (*Dalbergia odorifera*) seedlings [53]. In eleuthero (*Eleutherococcus senticosus*) seedlings, CO addition was found to promote not only fine root growth [54] as a unique driver, but also bioactive compounds in shoots in combination with LED spectrum [25]. All these findings together suggest a tendency that CO can be used as a regenerant from marine wastes and an environment-friendly reagent that can induce upregulation of bioactive compounds in more NWFP species. 

Black chokeberry (*Aronia melanocarpa* (Michx.) Elliott 1821) is a shrub from the rose family and native to boreal lands. Its natural populations are widely distributed in eastern North America, Scandinavia, and East Europe [55,56,57]. Rapeseed meal, microcrystalline cellulose, and various fruit pomace in its fruits are raw materials to make industrial biocomposites [58]. Extracts from its fruits have a significant dose of bioactive compounds, such as total phenolics (~12% dry weight [DW]) and proanthocyanidins (~7% DW) [59]. Its fruit juice was identified to have remarkable abilities in antioxidation [60] and immunomodulation [61]. A high content of anthocyanins contributes to a major composition of raw materials to produce natural dye [62]. Black chokeberry can acclimate to a wide range of biomes even with high survival rates on surface mines [63]. It is raised as a NWFP species in several cultivars that can well dwell in montane regions [56] and cut-over peatlands [64]. The purpose of the current study is to exhibit a comprehensive process of design, treatment, results, and mechanism to employ environment-friendly manipulations in the greenhouse to improve the fruit quality of black chokeberry seedlings. According to current findings, we hypothesized that artificial illumination using LED lighting can induce a better nutritional state and more qualified ripened fruits with higher bioactive compound content compared to controlled seedlings exposed to sunlight. Soils amended with SMR were assumed to contribute to the positive effect of LED lighting as a combined factor, while CO addition was hypothesized to increase the final accumulation of bioactive compounds in fruits.

## 2. Materials and Methods

### 2.1. Study Site and Experiment Layout

This study was conducted in a greenhouse constructed in a demonstration region of modern agricultural technologies (45°50′30″ N, 126°51′23″ E), Harbin, China. The greenhouse was used to demonstrate agronomic technologies for raising horticultural crops. Total planting area was 720 m^2^ arranged in a 12 m width and a 60 m length (Figure S1). Three-year old black chokeberry (*Aronia melanocarpa* (Michx.) Elliott 1821) seedlings were transplanted in a spacing of 1.5 m × 2 m (among individuals and planting lines, respectively) in the spring of 2019. Seven individuals were planted along a line leaving a buffering distance of 1.5 from either edge of the greenhouse to eliminate the marginal impact. Seedlings were planted in 29 lines leaving a buffering distance of 2 m from either end of the greenhouse. In this study, black chokeberry seedlings were cultured in a greenhouse constructed in a cold protective region. Factors were controlled across combined LED, SMR amendment, and CO addition in an equipped environment to test their potentially interactive effects on leaf parameters, antioxidant ability, and fruit quality. A total of 203 seedlings were transplanted and terminal buds and new shoots were pruned to keep stem length evenly about 2 m. Seedlings were fertilized by controlled release fertilizers (CRFs) in granules (N-P_2_O_5_-K_2_O, 14-14-14; micronutrients plus) (Osmocote, Scotts UK Ltd., Nottinghamshire, UK) at the rate of 12 g per plant. This fertilizer protocol has been well demonstrated to feed red raspberry (*Rubus idaeus* L.) cultivars with desired growth and reproduction [65,66,67]. Temperature in the micro-environment was controlled by collapsible cloths to stimulate wind flow and curtain coverage to obstruct sunlight radiation. During the experiment, temperature ranged between 19 °C and 31 °C with an average air relative humidity of ~63%. Seedlings were watered 1–3 times a week depending on soil moisture state. 

### 2.2. LED Light Treatment and Layout

Two LED light spectra were tested in this study; one red-color tinted and the other one blue-color tinted. The LEDs were designed in bulbs (Zhongke Rare Earth Inc., Plant Illumination Model, Changchun, China) whose spectra were adjusted by electric currents to power diodes emitting red, blue, and green lights (Table 1). Electric current for diodes were given through a transformer at 120 W that is embedded into the top of bulbs. LED bulbs were hanged at a height of 1.6 m aboveground. Photosynthetic photon flux density (PPFD) was measured to be 70–72, which were sampled on leaves subjected to the close side of LED bulbs (*n* = 4). LED bulbs were handed in two lines across the two ends of the greenhouse. Two pieces of black-out curtains were hanged beyond red and blue light tinted bulbs to avoid interaction between two different lights. Between the two lines of hanged LED bulbs, a line of seedlings was placed, which were just subjected to sunlight and taken as the control. These controlled seedlings were planted in a lined space in the width of 1.7 m. 

### 2.3. SMR Amendment Design and Layout

As shown in Figure S1, half of the seedlings were planted in soils that had been amended by SMR (Zhiluntuowei A&F S&T Inc., Changchun, China) and the other half in soils without amendment. A hand-held rotary tractor was used to shape cultural seeding beds in an area of 10.8 m^2^ (1.2 m in width and 9.0 m in length). SMRs were broadcasted on the surface along a seeding bed alternatively beside an adjacent bed receiving nothing. Seeding beds were shaped to a depth of 20 cm within which were SMR fully mixed with raw soils. The volume of SMR accounted for a quarter of that in a seeding bed which was identified to be an optimum proportion to benefit seedling growth and nutrient uptake [17,41,42]. After planting, all seeding beds were mulched using plastic black-out sheets (0.12 mm in thickness) to keep soil temperature warm and suppress weed growth. Four plots were randomly located in the greenhouse where initial soils and amended SMR were collected to determine their chemical properties. As shown in Table 2, initial soils had higher nitrate N content (~93 mg kg^−1^) and pH value (~6.2) than those in SMR (~13 mg kg-1 and ~5.4, respectively), but ammonium N content, phosphate P content, and electric conductance (EC) were lower in raw sols. Initial soils were collected by a hand-held drill (Ø: 5 cm) to a depth of 20 cm beneath the surface. SMR amendment was collected in the same volume at the same collecting plot.

### 2.4. CO Addition Treamtent

Six pairs of lined seedlings were chosen to receive additions of CO (Qishanbao, GlycoBio Co., Ltd., Dalian, China) (Figure S1). Seedlings in a cultural line were planted in controlled soils and the other line of seedlings were planted in SMR amended soils. These CO-treated seedlings included those not only subjected to two LED lights, but also sunlight. CO was sprayed to leaves of seedlings, which were covered by a plastic bag and subsequently received CO sprays. This treatment will ensure that CO spray won’t pollute adjacent seedling leaves. CO was sprayed at a concentration of 2 mg kg^−1^ (*w*/*w*) which has been well demonstrated to induce desired N and P utilization and stress resistance [18,51]. CO was added once a week throughout the whole experiment. 

### 2.5. Sampling and Analysis

In late August when seedlings were ripened to load fruits, fruits and leaves were sampled from two seedlings subjected to one of the 12 treatments combined with three lighting types (LED-red, LED-blue, sunlight), two amendment treatments (SMR vs. control), and two CO addition treatments (CO spray vs. no CO spray). Leaves and fruits were sampled from the of seedlings sides directly exposed to LED illumination. Height and root-collar diameter (RCD) were measured for the objective seedlings. Eight leaves and fruits were sampled from branches and bulked to establish a sampling pool. The average of eight samples was assigned as a location and four locations were randomly chosen as four replicates.

Five of the eight sampled leaves per seedling were randomly chosen, scanned to an image at 300 dots per inch (dpi), and analyzed to quantify projected area by WinRhizo software (Regent Instrument Inc., Calgary, Canada). Four groups of leaves per seedling were oven-dried at 70 °C for 72 h and measured for dry mass weight, and the other four groups of leaves were used for determining parameters based on fresh samples. Dried leaves were mixed and used for chemical analysis. Specific leaf area (SLA) was calculated as an equation [68]:

(1) $$\mathrm{SLA}=\frac{\mathrm{LA}}{\mathrm{LW}}$$

where LA is the single leaf area, and LW is the single leaf weight. 

Leaf concentrations of soluble sugars and starch were determined by a colorimetric method using a spectrophotometer (UV-Visible 8453, Agilent Technologies Inc., Santa Clara, CA, USA) at 490 nm [53]. A 0.2 g sample was digested in 5 mL digestion mixed by hydrogen peroxide and sulfuric acid [42]. Total N concentration was determined using the Kjeldahl method, and total P concentration was determined by the Molybdenum-anticolorimetry method. Leaf chlorophyl and protein contents were analyzed according to the methods adapted from Nosheen et al. [69]. Leaf glutamine synthetase (GS) activity was quantified using the method of Serapiglia et al. [70]. Briefly, a 0.5 g fresh leaf sample was homogenized in 5 mL extraction buffer (3.059 g Tris, 0.249 g MgSO_4_•7H_2_O, 0.309 g dithiothreitol, and 68.5 g sucrose; dissolution in 500 mL distilled water at 8.0 pH), and 0.7 mL of centrifuged samples was used for the quantification of GS activity at 540 nm. Leaf acid phosphatase (AC) activity was assayed using a method adapted from Kolari and Sarjala [71]. Briefly, a 0.1 g fresh leaf sample was ground in liquid N, centrifuged at 10,000 rpm for 10 min, added to the solution containing 0.2 mM CH_3_COONa and 0.6 mM p-nitrophenylphosphate, and measured at 405 nm. Assays on peroxidase (POD), superoxide dismutase (SOD), and catalase (CAT) were adapted from methods of Hussain et al. [72]. Fresh leaf samples were centrifuged at 15,000 rpm and supernatants were extracted by 400 µL guaiacol, 75 mM ethylenediaminetetraacetic acid, and 0.9 mL H_2_O_2_ plus 50 mM phosphate buffer for assays of POD, SOD, and CAT at 470 nm, 560 nm, and 240 nm, respectively. 

Fruit total phenolics were determined using the method of Podsędek et al. [73]. Dried fruits were ground and incubated at ambient temperature for 20 min. The content of total phenolics was measured as gallic acid equivalents using Folin-Ciocalteu reagent at 760 nm. Total proanthocayanidins were assayed using the method described by Rösch et al. [74]. This method quantified proanthocyanidins corresponding to acid depolymerization as an assessment of cyanidin equivalents (*ε*) using the equation:

(2) $$\varepsilon =\frac{17360L}{\mathrm{mol}\times \mathrm{cm}}$$

### 2.6. Statistics and Data Analysis

Statistics were finished using SAS software (ver. 9.4, SAS Inst., Cary, NC, USA). The normality of data was detected by Shapiro–Wilk test using the univariate procedure. All results passed the normal distribution test and variance was homogeneous. All data were analyzed in a split-block design wherein the main block was the variation of different lighting types, sub-blocks were soil amendment and CO addition. The random factor resulted from the random selection of replicated plots for sampling (*n* = 4). Analysis of variance (ANOVA) was used to detected potentially interactive effects of light × SMR × CO on leaf and fruit parameters. When a significant interaction was indicated (*α* = 0.05), results were compared in one-way ANOVA across combined treatments using Tukey test at 0.05 level of significance. Different means are marked by different letters for all comparisons. Nutritional statuses of seedlings subjected to contrasting conditions were diagnosed using the method of Salifu and Timmer [75].

## 3. Results and Discussion

### 3.1. Seedling Growth and Leaf Parameter

**Figure 1:**
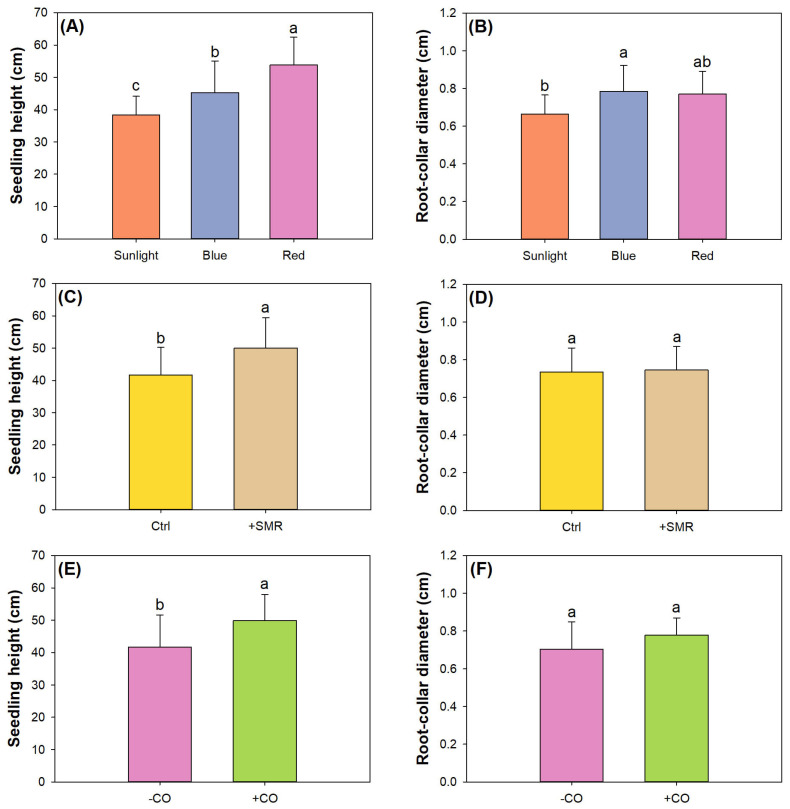
Growth of black chokeberry (*Aronia melanocarpa* (Michx.) Elliott 1821) exposed to separate light-type (sunlight, blue light-emitting diode (LED), red LED), chitosan oligosaccharide (CO), and spent mushroom residue (SMR) treatments: (**A**) light effect on seedling height; (**B**) light effect on root-collar diameter; (**C**) SMR effect on seedling height with unamended soils as the control (Ctrl); (**D**) SMR effect on root-collar diameter with unamended soils as the control (Ctrl); (**E**) CO effect on seedling height; (**F**) CO effect on root-collar diameter. Different letters mark significant difference according to Tukey test at 0.05 level.

Factors of light type, SMR amendment, and CO addition all had single effects on shoot height, but only light type had a significant single effect on RCD (Table 3). Compared to the sunlight treatment, the blue light LED increased height and RCD while the red light LED only increased height (Figure 1A,B). The promotion of the height growth of plants exposed to spectra enriched with red light has also been reported in other studies [24,53]. This effect was evaluated to be positive for plants whose shoot growth was achieved by artificial lighting to harvest dry mass. The null effect of red light exposure on diameter growth was also reported in tree crops [43,76]. This was evaluated to be a by-product-result that shapes a seedling as a slender stock which has low quality although height may be significant. However, our results reveal that exposure to blue light can promote both height and diameter increments. 

Compared to controlled soils, SMR amended soils increased seedling height but not RCD (Figure 1C,D). Our study employed SMR amended to soils in a proportion of 25% in volume, which was also found to promote height growth of pepper (*Capsicum annum* L.) [42] but still failed to affect RCD in fragrant rosewood (*Dalbergia odorifera*) seedlings [17]. Again, CO addition also increased height without a significant effect on RCD (Figure 1E,F).

**Figure 2:**
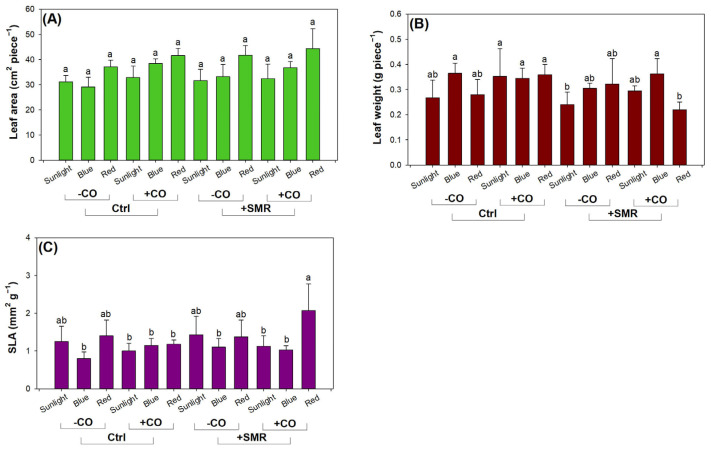
Leaf morphology and weight of black chokeberry (*Aronia melanocarpa* (Michx.) Elliott 1821) exposed to combined light-type (sunlight, blue light-emitting diode (LED), red LED), chitosan oligosaccharide (CO), and spent mushroom residue (SMR) treatments: (**A**) combined effects of LED, CO, and SMR on leaf area; (**B**) combined effects of LED, CO, and SMR on leaf weight; (**C**) combined effects of LED, CO, and SMR on specific leaf area. Different letters mark significant difference according to Tukey test at 0.05 level.

Factors of light type, SMR amendment, and CO addition had interactive effects on single leaf weight and SLA (Table 3). Light type and CO addition had single effects on LA. LA was larger in red light (41.20 ± 4.66 cm^2^) compared to that in sunlight (32.07 ± 3.75 cm^2^), but LA was not changed in blue light (34.38 ± 4.34 cm^2^) from that in sunlight (Figure 2A). A similar effect of LED spectrum enriched in red light on leaf area was also reported on cucumber (*Cucumis sativus* L. cv. Yuexiu no. 3) [77]. The enlargement of leaf area to touch light resource is a strategy by which red light can elevate light assimilation and use it for height growth. CO addition increased LA (37.76 ± 5.34 cm^2^) compared to no additive treatment (34.01 ± 4.81 cm^2^). This agrees with results found on coffee (*Coffea canephora* var *Robusta*) [78]. 

Sunlight did not result in different LW from LED lighting in any treatments combining SMR amendment and CO addition (Figure 2B). Seedlings exposed to sunlight without CO addition in SMR amended soils had lower LW than those with CO addition in controlled soils. These suggested that CO addition promoted dry mass production and retained in leaves at a higher rate than SMR amendment. The red light reduced LW more than the blue light in seedlings only dually with CO addition and SMR amendment. 

Seedlings subjected to red light with CO addition in SMR amended soils showed higher SLA than those in most other combined treatments (Figure 2C). These findings result from increased LW in red light. However, seedlings exposed to sunlight or red light without CO addition showed SLAs that had no difference with seedlings in other treatments. Even so, we cannot conclude that red light can improve the efficacy to assimilate light resource by adjusting input to leaf morphology. The effect of red light relies heavily on the joint contributions of SMR amendment and CO addition. 

### 3.2. Nutrient Uptake and Status Diagnosis

**Figure 3:**
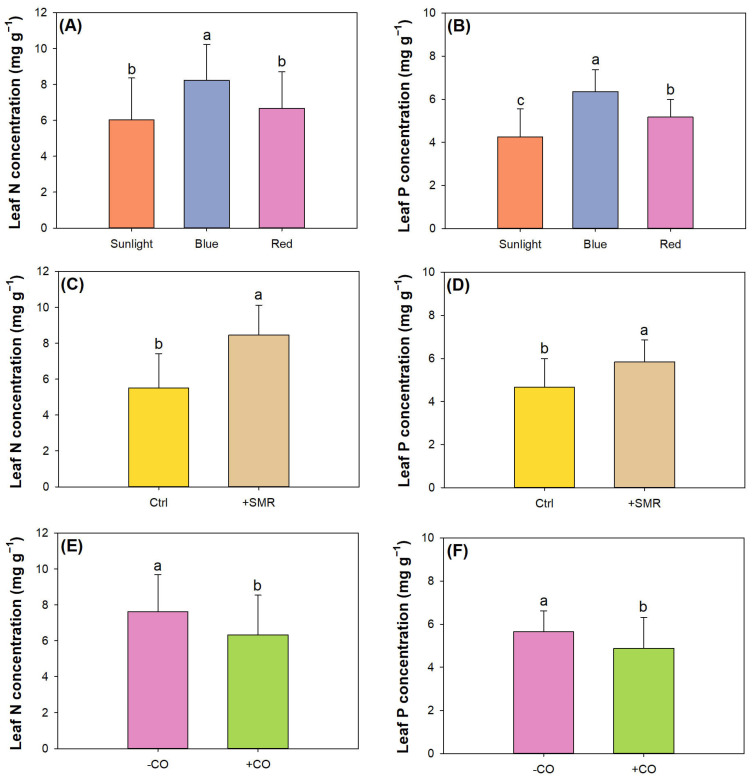
Leaf nitrogen (N) and phosphorus (P) concentrations in black chokeberry (*Aronia melanocarpa* (Michx.) Elliott 1821) exposed to separate light-type (sunlight, blue light-emitting diode (LED), red LED), chitosan oligosaccharide (CO), and spent mushroom residue (SMR) treatments: (**A**) light effect on leaf N concentration; (**B**) light effect on leaf P concentration; (**C**) SMR effect on leaf N concentration with unamended soils as the control (Ctrl); (**D**) SMR effect on leaf P concentration with unamended soils as the control (Ctrl); (**E**) CO effect on leaf N concentration; (**F**) CO effect on leaf P concentration. Different letters mark significant difference according to Tukey test at 0.05 level.

Factors of light type, SMR amendment, and CO addition all had single effects on leaf total N and P concentrations (Table 4). In addition, light type and CO addition also had an interactive effect on leaf total P concentration. Blue light increased leaf total N and P concentrations compared to sunlight, and red light only increased leaf total P concentration compared to sunlight (Figure 3A,B). Red light was also found to be less effective to promote N and P concentrated in leaves of *Ficus hirta* [24] and *Quercus variabilis* Blume seedlings [19] compared to blue and green lights. Our blue light was tinted by more blue and green colors of lights (proportions of 15.3% and 64.9%, respectively). Hence, the positive effect of blue light on N and P concentrations should be attributed to a high proportion of combined blue and green lights. 

SMR amendment increased leaf N and P concentrations (Figure 3C,D), and CO addition decreased these concentrations (Figure 3E,F). SMR had a nature of higher ammonium N and available P compared to raw soils, which benefitted N and P availabilities [17,41,42]. CO stimulated seedling growth and leaf area but did not provide substantial inputs of N or P. Therefore, nutritional concentration can unlikely be promoted by CO addition. Leaf P concentration was not changed in seedlings exposed to different light types without CO addition (average: 5.64 mg g^−1^). Seedlings with CO addition, however, had higher leaf P concentration in blue light (6.58 ± 0.80 mg g^−1^) compared to those in sunlight (3.52 ± 1.38 mg g^−1^). 

**Figure 4:**
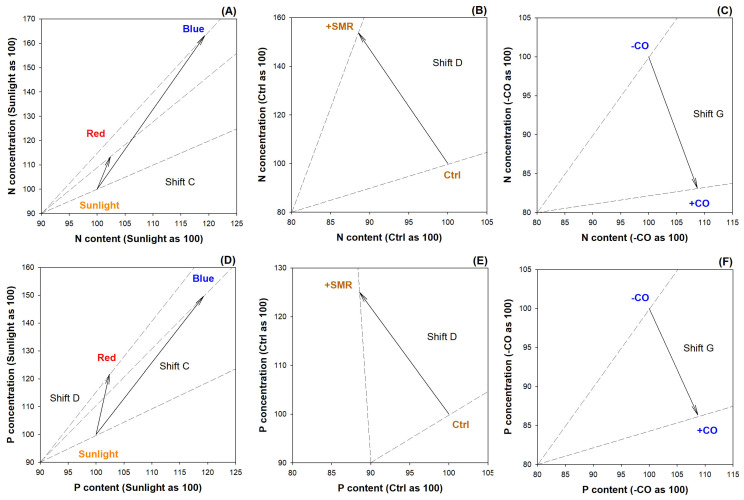
Vector diagnoses of leaf nitrogen (N) and phosphorus (P) statuses in black chokeberry (*Aronia melanocarpa* (Michx.) Elliott 1821) exposed to separate light-type (sunlight, blue light-emitting diode (LED), red LED), chitosan oligosaccharide (CO), and spent mushroom residue (SMR) treatments: (**A**) diagnosis of leaf N status exposed to different light types; (**B**) diagnosis of leaf N status exposed to SMR amendment; (**C**) diagnosis of leaf N status exposed to CO addition; (**D**) diagnosis of leaf P status exposed to different light types; (**E**) diagnosis of leaf P status exposed to SMR amendment; (**F**) diagnosis of leaf P status exposed to CO addition. Abbreviations: Red, red light LED; Blue, blue light LED; +SMR, SMR amendment; Ctrl, no SMR amendment; -CO, no CO addition; +CO, with CO addition. Shifts and interpretations are adapted from Salifu and Timmer [75]: Shift C, nutrient deficiency; Shift D, luxury consumption; Shift G, nutrient depletion.

Both types of LED lights (red and blue colors) increased leaf biomass, nutritional (N and P) contents, and concentrations relative to sunlight, which were characterized as a nutrient limiting state for seedlings tested at sampling, and LED light induced an effect to counter this negative status (Figure 4A,D). Compared to controlled soils, SMR amendment increased leaf biomass, but it did not increase nutritional (N and P) concentrations to significant levels (Figure 4B,E). Therefore, the increase in nutrient content accorded with the growth of biomass. These changes were characterized as a luxury consumption state. In contrast, nutritional (N and P) concentrations did not decrease significantly and the decline of biomass drove the decrease in nutrient content, which was characterized as nutrient depletion (Figure 4C,F). 

### 3.3. Photosynthetic Pigment and Nutrient Assimilation

**Figure 5:**
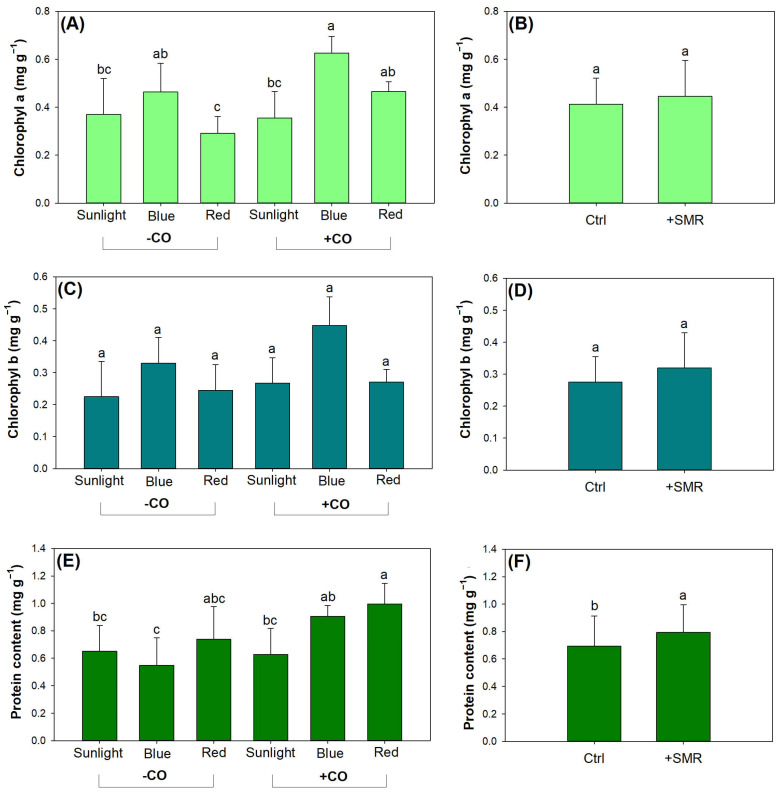
Leaf chlorophyl and protein contents in black chokeberry (*Aronia melanocarpa* (Michx.) Elliott 1821) exposed to combined light-type (sunlight, blue light-emitting diode (LED), red LED), chitosan oligosaccharide (CO), and spent mushroom residue (SMR) treatments: (**A**) combined CO and light effects on chlorophyl a content; (**B**) SMR effect on chlorophyl a content with unamended soils as the control (Ctrl); (**C**) combined CO and light effects on chlorophyl b content; (**D**) SMR effect on chlorophyl b content with unamended soils as the control (Ctrl); (**E**) combined CO and light effects on protein content; (**F**) SMR effect on protein content with unamended soils as the control (Ctrl). Different letters mark significant difference according to Tukey test at 0.05 level.

Light type and CO addition had an interactive effect on chlorophyl a content and either factor had a single effect on chlorophyl b content (Table 4). Compared to sunlight, blue light increased chlorophyl a content only in seedlings with CO addition, but red light failed to increase chlorophyl a content (Figure 5A). This was also confirmed by Gao et al. [19] on *Q. variabilis* seedlings. In seedlings without CO addition, red light reduced chlorophyl a content compared to blue light. SMR did not change chlorophyl a content (Figure 5B). Chlorophyl b content was higher in blue light (0.39 ± 0.09 mg g^−1^) compared to that in sunlight (0.25 ± 0.09 mg g^−1^) and in red light (0.26 ± 0.06 mg g^−1^) (Figure 5C). CO addition increased chlorophyl b content from 0.33 ± 0.10 mg g^−1^ to 0.27 ± 0.09 mg g^−1^, but SMR amendment did not cause any change in chlorophyl b content (Figure 5D). 

Red light increased leaf protein content compared to sunlight in seedlings with CO addition, but results were not the case for seedlings without CO addition (Figure 5E). Seedlings in blue light without CO addition reduced leaf protein in blue and red lights with CO addition. SMR amendment increased leaf protein content (Figure 5F). 

**Figure 6:**
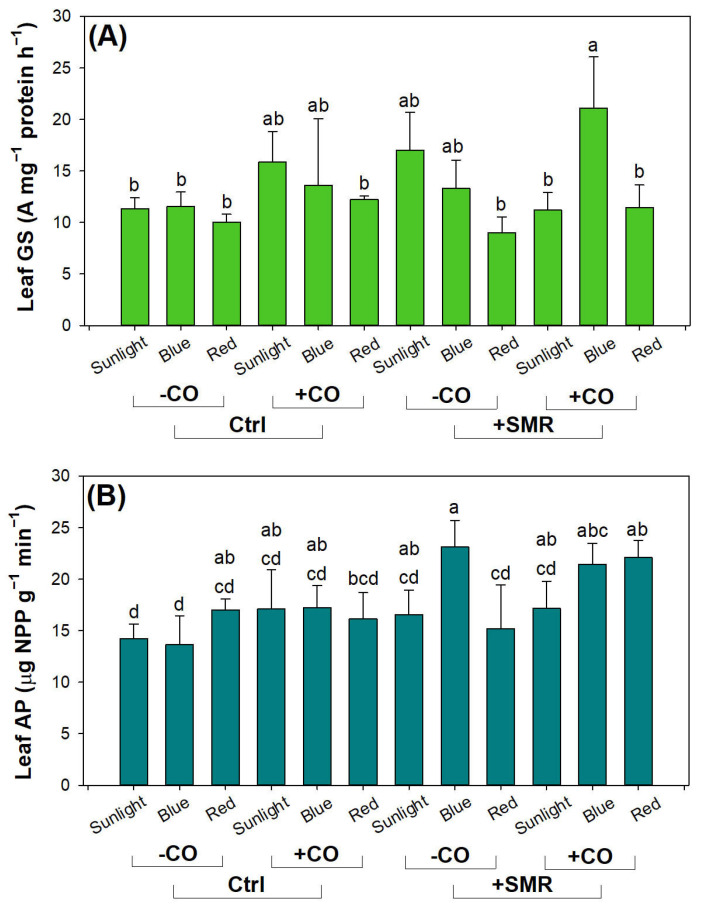
Leaf glutamine synthetase (GS) and acid phosphate (AP) activities in black chokeberry (*Aronia melanocarpa* (Michx.) Elliott 1821) exposed to combined light-type (sunlight, blue light-emitting diode (LED), red LED), chitosan oligosaccharide (CO), and spent mushroom residue (SMR) treatments: (**A**) combined light, SMR, and CO effects on GS activity; (**B**) combined light, SMR, and CO effects on AP activity. Different letters mark significant difference according to Tukey test at 0.05 level.

Both activities of GS and AP showed significant responses to combined light type, SMR amendment, and CO addition (Table 4). Leaf GS activity was higher in blue light than in sunlight only in seedlings with CO addition in soils amended with SMR (Figure 6A). GS activity in this combined treatment was higher than that in red light in any other combined treatments. The upregulation of GS activity in blue light was attributed to an increase of the responsiveness to phytochrome [79]. Leaf AP activity was higher in blue light than in red light without CO addition in SMR amended soils (Figure 6B). In seedlings without CO addition, blue light also increased leaf AP activity in SMR amended soils more than controlled soils. SMR amendment inputs a significant dose of available P to soils, which was absorbed by seedlings and strengthened by blue light [28]. Promoted GS and AP activities accord with N and P availabilities, respectively, which had both been reinforced by SMR amendment. 

### 3.4. Antioxidant Activity

**Figure 7:**
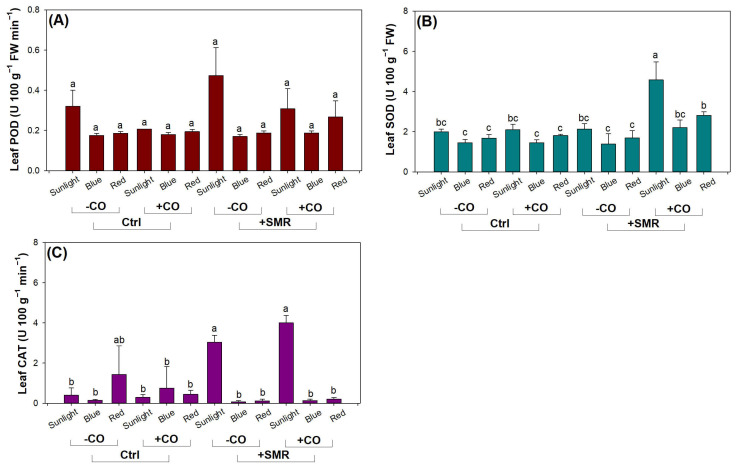
Leaf antioxidant activities in black chokeberry (*Aronia melanocarpa* (Michx.) Elliott 1821) exposed to combined light-type (sunlight, blue light-emitting diode (LED), red LED), chitosan oligosaccharide (CO), and spent mushroom residue (SMR) treatments: (**A**) combined light, SMR, and CO effects on peroxidase (POD) activity; (**B**) combined light, SMR, and CO effects on superoxide dismutase (SOD) activity; (**C**) combined light, SMR, and CO effects on catalase (CAT) activity. Different letters mark significant difference according to Tukey test at 0.05 level.

Light type had interactive effects with SMR and CO addition on POD activity (Table 5). Seedling in soils with SMR amendment in sunlight had higher POD activity (0.39 ± 0.13 U 100 g^−1^ FW min^−1^) than that (averaged 0.20 ± 0.04 U 100 g^−1^ FW min^−1^) in other combined treatments (Figure 7A). Seedling without CO addition in sunlight had higher POD activity (0.40 ± 0.13 U 100 g^−1^ FW min^−1^) than that (averaged 0.21 ± 0.03 U 100 g^−1^ FW min^−1^) in other combined treatments. Therefore, sunlight in our study is not intensified enough to feed black chokeberry seedlings with desired light resources and induce antioxidant defense. 

Both SOD and CAT activities were significantly responsive to combined light, SMR, and CO treatments (Table 5). Seedlings in sunlight with CO addition in SMR amended soils had the highest leaf SOD activity among all combined treatments (Figure 7B). Red light with CO addition in SMR amendment resulted in higher SOD activity than that in red and blue lights in any other combined treatments. In soils amended with SMR, sunlight resulted in higher CAT activities with or without CO addition compared to any other combined treatments except for that in red light without CO addition in controlled soils (Figure 7C). These findings suggest that CO addition can be a trigger to induce antioxidant defense, which has also been confirmed in Cucumber (*C. sativus* L.) [80] and Buddhist pine (*Podocarpus macrophyllus*) [18].

### 3.5. Fruit Quality Assessment

**Figure 8:**
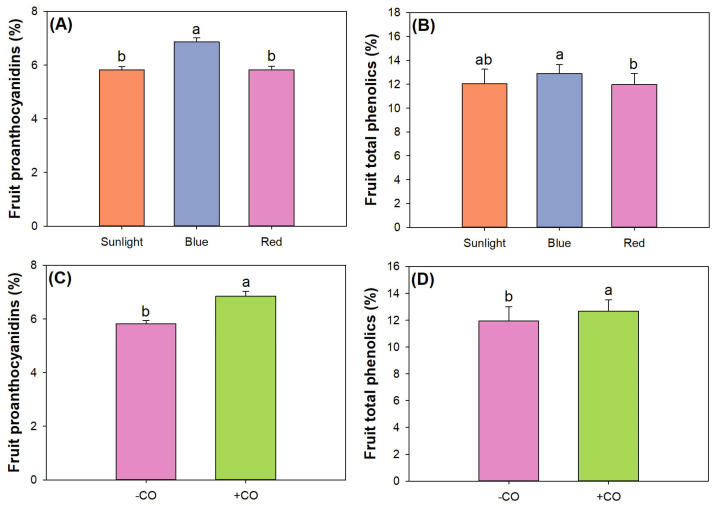
Quality assessment of fruits in black chokeberry (*Aronia melanocarpa* (Michx.) Elliott 1821) exposed to separate light-type (sunlight, blue light-emitting diode (LED), red LED), chitosan oligosaccharide (CO), and spent mushroom residue (SMR) treatments: (**A**) light effect on leaf proanthocyanidins; (**B**) light effect on total phenolics in fruits; (**C**) CO effect on fruit proanthocyanidins; (**D**) CO effect on total phenolics in fruit. Different letters mark significant difference according to Tukey test at 0.05 level.

We employed a split-block model to lay out the experiment with three factors, but rare of them had any interactive effects to impact fruit quality formation in black chokeberry seedlings. Light type and CO addition had single effects on fruit contents of proanthocyanidins and total phenolics, but SMR amendment had no effect (Table 5). Blue light increased fruit proanthocyanidin content relative to sunlight, but red light did not cause any increase (Figure 8A). Neither blue light nor red light resulted in higher fruit content of total phenolics compared to sunlight, and blue light resulted in higher total phenolics than red light (Figure 8B). In accordance with our results, Zhang et al. [81] also reported that proanthocyanidin biosynthesis was promoted in strawberry exposed to combined red and blue lights. Our light spectra from LED presented a mixture across multiple wavelengths of lights rather than any single light spectrum. Our blue light spectrum was a combination of lights of varied spectra where red light accounted for a proportion lower than 50%. In addition, our blue light resulted in higher proanthocyanidins and total phenolics in fruits than red light. To our knowledge, rare evidence can be found from current literature as a reference to this part of our results.

CO addition increased both contents of proanthocyanidins and total phenolics (Figure 8C,D). These results clearly indicated that CO addition can promote fruit contents of proanthocyanidins and total phenolics. CO addition was also reported to increase the content of phenolic substance in arugula (*Eruca vesicaria* ssp. *sativa*) [82]. The increase of proanthocyanidin content with CO addition resulted from their conjugation [83]. 

## 4. Conclusions

This study demonstrates a comprehensive instance to raise black chokeberry seedlings with multiple environment-friendly factors controlled. Our results confirm that the use of greenhouse can be a nature/industry-based solution to obtain high content of natural proanthocyanidins from fruits by employing combined blue light LED lighting and CO addition. This combined treatment can also promote the efficacy to absorb and assimilate N and increase the probability to harvest total phenolics. The use of SMR did not modify fruit quality. SMR amendment can reinforce N and P availabilities to induce their steady-state uptakes by plants. Hence, SMR may not be necessary recommended unless the goal of plant breeding program is to consider inherent nutrient reserve establishment for over-year transplant. In our study, we do not recommend using SMR in practical work because black chokeberry seedlings are planned to be established as a continuous producer of natural bioactive compounds. 

Our study is a frontier to test multiple environment-friendly factors controlled in a greenhouse for the culture of black chokeberry seedlings. Further work is firstly suggested to test our results in more places to eliminate the potential impacts of regional meteorological conditions on fruit quality. Furthermore, our study reveals an initial trial testing combined treatments of light, SMR, and CO. Rates and levels to employ these three manipulations can be extended in further studies to obtain a more precise dose-dependent cultural protocol. Finally, we suggest that future work employ soil-free substrates to culture potted black chokeberry seedlings to fully diminish the possible effects of raw soils. Thus, soils were subjected to previous protocols using pesticides, herbicides, and the intensive input of chemical fertilizers. Peat or decomposed spent mushroom residues deserve future tests. 

## Figures and Tables

**Table 1:** Properties of contrasting lights (red-color-tinted vs. blue-color-tinted) in light emitting diodes (LEDs) for the culture of black chokeberry (*Aronia melanocarpa* (Michx.) Elliott 1821) seedlings in a greenhouse.

Light Type	Current Composition ^1^	PPFD ^2^ (µ mol^−1^ m^−2^ s^−1^)	Spectrum Composition ^3^
Red-tinted	70% R and 10% B + G	72.39 ± 1.99	69.4%:30.2%:0.4% ^4^
Blue-tinted	30% R and 100% B + G	70.81 ± 2.22	15.3%:64.9%:19.8%

^1^ the composition of currents to adjust intensities of red-color-tinted and blue-color-tinted LED lights; R, red light; B, blue light; G, green light; ^2^ PPFD, photosynthetic photon flux density; ^3^ Spectrum composition was quantified about 45 cm beneath the LED illuminators; ^4^ proportions was arranged in an order of R, G, and B.

**Table 2:** Chemical properties in soils and spent mushroom residues (SMRs) for the culture of black chokeberry (*Aronia melanocarpa* (Michx.) Elliott 1821) seedlings in a greenhouse.

Source	NH_4_^+^-N ^1^ (mg kg^−1^)	NO_3_^−^-N ^2^ (mg kg^−1^)	PO_4_^3−^-P ^3^ (mg kg^−1^)	pH	EC ^4^ (dS m^−1^)
SMR	11.59 ± 1.21 a ^5^	12.47 ± 2.15 b	773.57 ± 159.50 a	5.36 ± 0.29 b	2.30 ± 0.34 a
Soil	3.97 ± 3.05 b	93.34 ± 9.39 a	1.85 ± 0.29 b	6.19 ± 0.56 a	0.13 ± 0.04 b
*F* value	18.73	245.26	81.52	6.14	137.41
*p* value	0.0049	<0.0001	0.0001	0.0479	<0.0001

^1^ NH_4_^+^-N, ammonium nitrogen content; ^2^ NO_3_**^−^**-N, nitrate nitrogen content; ^3^ PO_4_^3−^-P, phosphate phosphorus content; ^4^ EC, electric conductance; ^5^ different letters along a column indicate significant difference at 0.05 level according to Tukey test.

**Table 3:** *F* values from analysis of variance (ANOVA) of combined factors of lighting spectra (Light), soil amendment (Soil), and chitosan oligosaccharide (CO) addition on growth, leaf morphology, and leaf weight in black chokeberry (*Aronia melanocarpa* (Michx.) Elliott 1821) seedlings.

Source	Height	RCD ^1^	LA ^2^	LW ^3^	SLA ^4^
Light	16.18 *** ^5^	3.88 *	17.07 ***	3.55 *	6.96 **
Soil	13.96 ***	0.07	1.49	4.07	4.88 *
CO	13.57 ***	3.74	8.01 **	1.93	0.03
Light × Soil	0.03	0.15	0.67	0.20	1.16
Light × CO	1.34	0.07	1.29	1.63	2.03
Soil × CO	1.12	4.12	1.18	1.46	0.58
Light × Soil × CO	0.14	1.81	0.30	4.12 *	3.28 *

^1^ RCD, root-collar diameter; ^2^ LA, leaf area; ^3^ LW, leaf weight; ^4^ SLA, specific leaf area; ^5^ number of asterisks indicates degree of significance: *, *p* < 0.05; **, *p* < 0.01; ***, *p* < 0.001.

**Table 4:** *F* values from analysis of variance (ANOVA) of combined factors of lighting spectra (Light), soil amendment (Soil), and chitosan oligosaccharide (CO) addition on nutritional and physiological parameters in leaves of black chokeberry (*Aronia melanocarpa* (Michx.) Elliott 1821) seedlings.

Source	TN ^1^	TP ^2^	Chla ^3^	Chlb ^4^	Protein ^5^	GS ^6^	AP ^7^
Light	6.40 ** ^8^	20.10 ***	12.21 ***	12.78 ***	8.87 ***	7.28 **	3.47 *
Soil	32.32 ***	18.40 ***	1.00	3.05	5.28 *	2.25	17.56 ***
CO	6.12 *	8.01 **	10.37 **	5.91 *	19.43 ***	5.51 *	5.53 *
Light × Soil	0.23	1.03	0.79	0.96	6.26 **	3.14	4.75 *
Light × CO	1.80	5.07 *	3.38 *	1.21	6.51 **	2.93	0.55
Soil × CO	0.07	0.34	0.36	1.20	16.43 ***	0.59	0.01
Light × Soil × CO	2.20	0.48	0.22	0.67	0.62	6.28 **	6.04 **

^1^ TN, total nitrogen (N) concentration; ^2^ TP, total phosphorus (P) concentration; ^3^ Chla, chlorophyl a content; ^4^ Chlb, chlorophyl b content; ^5^ Protein, leaf protein content; ^6^ GS, glutamine synthetase activity; ^7^ AP, acid phosphatase activity; ^8^ number of asterisks indicates degree of significance: *, *p* < 0.05; **, *p* < 0.01; ***, *p* < 0.001.

**Table 5:** *F* values from analysis of variance (ANOVA) of combined factors of lighting spectra (Light), soil amendment (Soil), and chitosan oligosaccharide (CO) addition on antioxidant enzymes’ activities in leaves and fruit quality of black chokeberry (*Aronia melanocarpa* (Michx.) Elliott 1821) seedlings.

Source	POD ^1^	SOD ^2^	CAT ^3^	Proa ^4^	Total Phenolics
Light	23.36 *** ^5^	30.22 ***	14.30 ***	13.45 ***	3.65 *
Soil	8.59 **	39.29 ***	65.12 ***	0.39	2.07
CO	2.15	45.64 ***	1.83	25.36 ***	5.53 *
Light × Soil	3.91 *	6.68 ***	9.42 ***	0.23	1.35
Light × CO	9.05 ***	5.02 *	23.90 ***	0.33	0.13
Soil × CO	0.07	36.87 ***	2.40	0.72	0.50
Light × Soil × CO	0.89	4.47 *	21.49 ***	0.36	0.59

^1^ POD, peroxidase activity; ^2^ SOD, superoxide dismutase activity; ^3^ CAT, catalase activity; ^4^ Proa, fruit proanthocyanidins content; ^5^ number of asterisks indicates degree of significance: *, *p* < 0.05; **, *p* < 0.01; ***, *p* < 0.001.

## Data Availability

Not applicative.

## Supplementary Materials

The following supporting information can be downloaded at: https://www.mdpi.com/article/10.3390/plants12030604/s1, Figure S1: Layout of the experiment conducted in a greenhouse to culture Black chokeberry (*Aronia melanocarpa* (Michx.) Elliott 1821) Exposed to Combined Light-Type, Chitosan Oligosaccharide, and Spent Mushroom Residue Treatments.
