# Supplementary material for: Natural Bioactive Substances in Fruits of Aronia melanocarpa (Michx.) Elliott Exposed to Combined Light-Type, Chitosan Oligosaccharide, and Spent Mushroom Residue Treatments
Source: Plants (Basel). 2023 Jan 30;12(3):604. doi: 10.3390/plants12030604 (PMC9919629; doi:10.3390/plants12030604)
Supplement: Supplementary file 1 [file plants-12-00604-s001.zip › plants-2176881-supplementary.pdf]

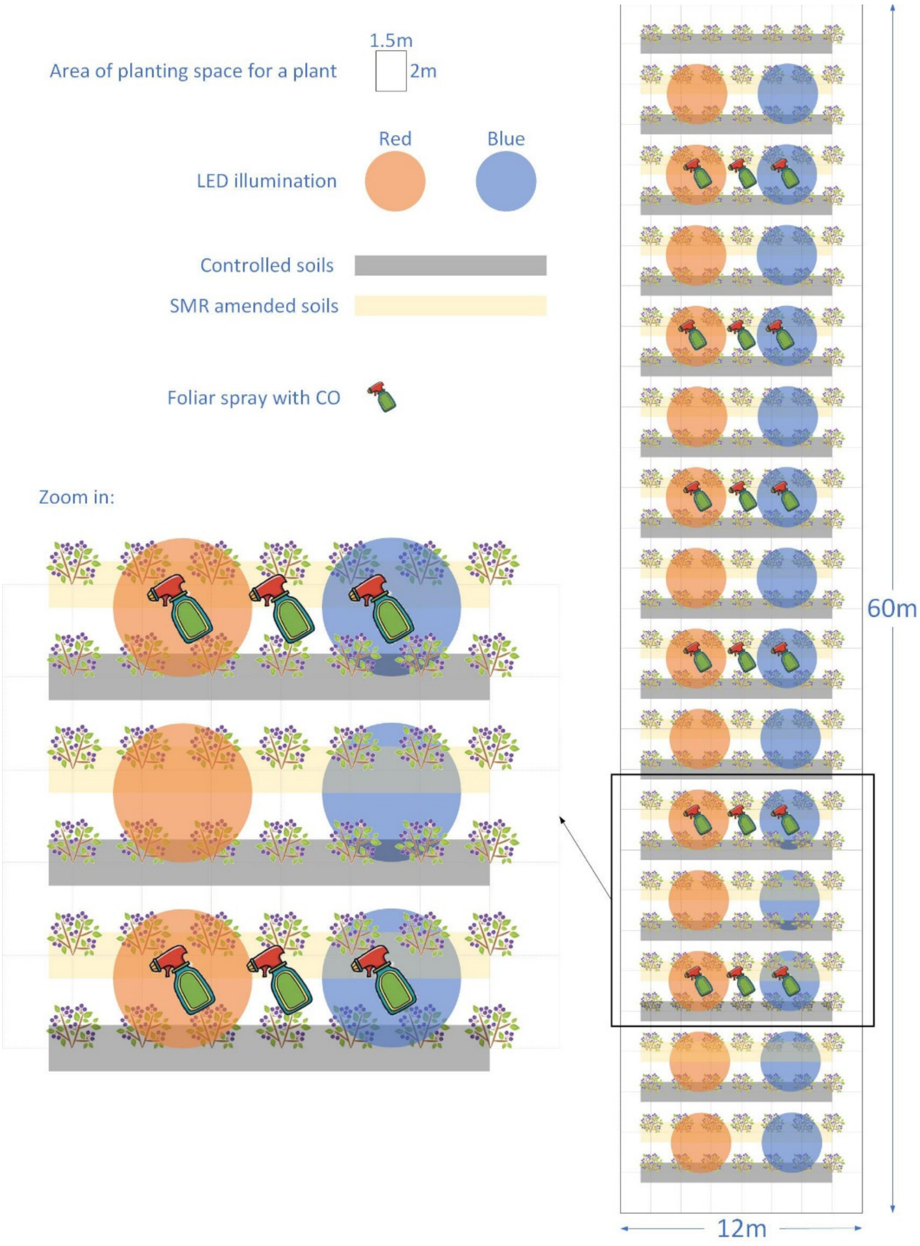

Figure S1. Layout of the experiment conducted in a greenhouse to culture Black chokeberry (*Aronia melanocarpa* (Michx.) Elliott 1821) Exposed to Combined Light-Type, Chitosan Oligosaccharide, and Spent Mushroom Residue Treatments.
